# The Profile of Retinal Ganglion Cell Death and Cellular Senescence in Mice with Aging

**DOI:** 10.3390/ijms26125436

**Published:** 2025-06-06

**Authors:** Wen-Ying Wang, Xin Bin, Yanxuan Xu, Si Chen, Shuyi Zhou, Shaowan Chen, Yingjie Cao, Kunliang Qiu, Tsz Kin Ng

**Affiliations:** 1 Joint Shantou International Eye Center of Shantou University and The Chinese University of Hong Kong, Shantou 515041, China; 2 Department of Ophthalmology and Visual Sciences, The Chinese University of Hong Kong, Hong Kong, China

**Keywords:** retinal ganglion cells, aging, cellular senescence, cell death

## Abstract

Older age is a risk factor for glaucoma, in which progressive retinal ganglion cell (RGC) loss leads to visual field defects and irreversible visual impairment and even blindness. We recently identified the involvement of cellular senescence in RGC cell death post-optic nerve injury. Here we further aimed to delineate the profile of RGC survival in mice with aging, a physiological process with increasing cellular senescence. The numbers of senescent cells in the ganglion cell layer (GCL) significantly and progressively increased starting at 8 months of age. Yet, significant reduction of ganglion cell complex layer thickness began in the 10-month-old mice, and significant reduction in the number of RGCs began in the 12-month-old mice as compared to the 2-month-old mice. Meanwhile, pyroptosis and ferroptosis markers as well as cellular senescence-related cell cycle arrest proteins p15^Ink4b^, p16^Ink4a^, p21^Cip1^, and p53 were significantly and progressively increased in GCL. In contrast, there were no significant changes in dendritic field, complexity, and branches with increasing ages. Comparing between the 2- and 16-month-old mouse retinas, the differentially expressed genes were involved in the pathways of neurodegeneration, innate immunity, and mitochondrial ATP synthesis. In summary, this study revealed the gradual increase in senescent cells as well as pyroptosis and ferroptosis with progressive RGC reduction in mice with aging. Cellular senescence and the related cell death pathways are potential targets for age-related RGC reduction.

## 1. Introduction

Glaucoma is a leading cause of irreversible visual impairment and blindness worldwide, affecting 76 million 40 to 80-year-old people globally in 2020 and predictably 112 million people in 2040 [1]. It is characterized by progressive retinal ganglion cell (RGC) death, retinal nerve fibre layer (RNFL) thinning, optic disc cupping, and visual field defects with or without intraocular pressure (IOP) elevation [2]. At present, current clinical treatments for glaucoma are still limited to the IOP lowering medications and surgeries [3]. In-depth understanding of the pathophysiology of glaucoma can help to identify additional treatment targets and strategies.

Apart from too high IOP, older age has also been indicated as one of the major risk factors for adult-onset glaucoma [4], and glaucoma risk increases with age [5]. In human, 0.50%/year of age-related RGC loss and 0.27%/year of age-related RNFL thinning have been reported [6]. For the animal studies, less RGCs were found in older mice and rats as compared to the younger ones [7,8,9]. In contrast, some studies reported no significant changes in RGCs with increasing ages in mice and rats [10,11,12]. Therefore, it is still controversial whether the number of RGCs would re-duce with increasing ages. Confirmation of the profile of RGC changes with aging can consolidate the physiological process of RGC survival regulation during aging.

For the mechanisms of RGC survival regulation, we previously delineated the longitudinal profile of different modes of cell death in mouse retina after optic nerve injury [13], and we recently demonstrated that cellular senescence is involved in RGC survival regulation in rodents with optic nerve injury [14]. We intend to further confirm the association of RGC death and cellular senescence in additional models and conditions. Considering that increasing ages physio-logically accompanies with the increase in cellular senescence [15], we, in this study, aimed to determine the profile of RGC changes and cellular senescence in mice with aging. In addition, the potential underlying mechanisms were also delineated by cell death and transcriptomic analyses. This section may be divided by subheadings. It should provide a concise and precise description of the experimental results, their interpretation, as well as the experimental conclusions that can be drawn.

## 2. Results

### 2.1. Changes of Senescence-Associated β-Galactosidase Expression in Mice During Aging

**Figure 1:**
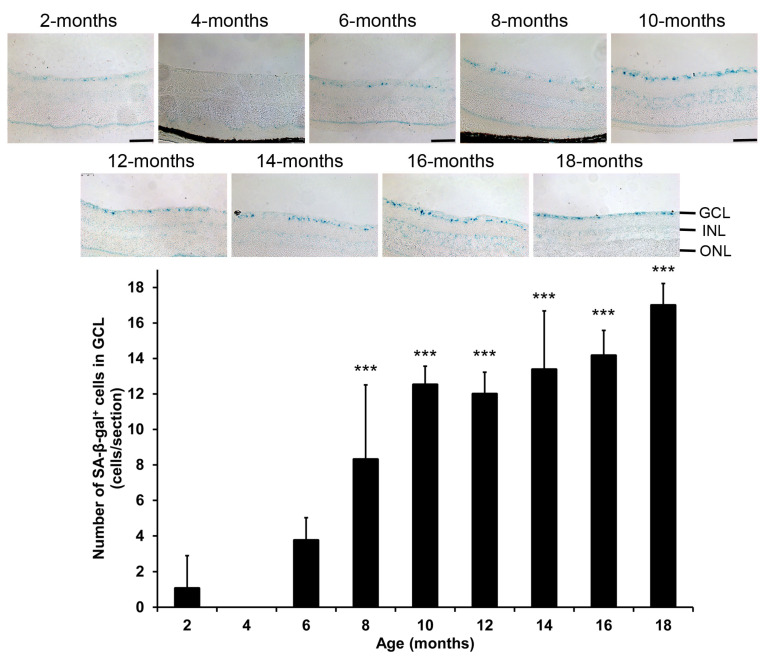
Changes of senescence-associated β-galactosidase expression in mice during aging. Staining and quantification of senescence-associated β-galactosidase (SA-βgal) activity (blue) in ganglion cell layer (GCL) of the 2-to 18-month-old mice. Scale bar: 100 μm. INL: inner nuclear layer; ONL: outer nuclear layer. Data were presented as mean ± standard deviation and compared by one-way analysis of variance with post hoc LSD test. *** *p* < 0.001 as compared to the 2-month-old mice.

Before assessing the changes of RGCs during aging, we first aimed to confirm the association of cellular senescence with increasing ages by the analysis of the senescent cells. The SA-βgal staining showed that the number of SA-βgal-positive cells in GCL of the 4- (0.00 ± 0.00 cells, *p* = 0.425) and 6-month-old mice (3.75 ± 1.27 cells, *p* = 0.063) showed no significant differences as compared to that of the 2-month-old mice (1.06 ± 1.84 cells; Figure 1 and Figure S1). Instead, the numbers of SA-βgal-positive cells in GCL were significantly and progressively increased in the 8- (8.31 ± 4.20 cells, *p* < 0.001), 10 (12.52 ± 1.04 cells, *p* < 0.001), 12- (12.00 ± 1.22 cells, *p* < 0.001), 14- (13.38 ± 3.30 cells, *p* < 0.001), 16- (14.17 ± 1.42 cells, *p* < 0.001), and 18-month-old mice (17.00 ± 1.22 cells, *p* < 0.001) as compared to that in the 2-month-old mice, confirming that there is a progressive elevation of senescent cells in mouse retinas with increasing ages, starting from 8 months of age.

### 2.2. Changes of Ganglion Cell Complex Layer Thickness in Mice During Aging

**Figure 2:**
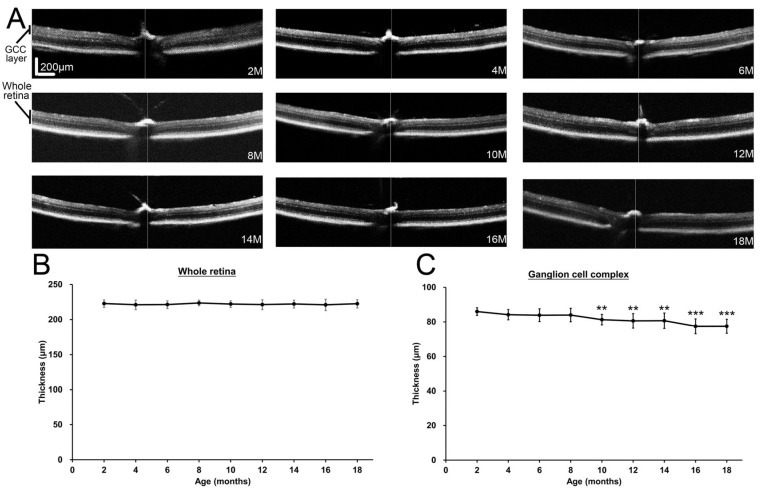
Changes of ganglion cell complex layer thickness in mice during aging. (**A**) Spectral domain-optical coherence tomography analysis on cross-sectional retina of the 2- to 18-month-old (M) mice in vivo. The thicknesses of (**B**) whole retina and (**C**) ganglion cell complex (GCC) layer (composing of retinal nerve fibre layer, ganglion cell layer, and inner plexiform layer) were measured. Vertical and horizontal scale bars: 200 μm. Data were presented as mean ± standard deviation and compared by one-way analysis of variance with post hoc LSD test. ** *p* < 0.01; *** *p* < 0.001 as compared to the 2-month-old mice.

With the confirmation of the association of cellular senescence with increasing ages in mouse retina, we next evaluated the longitudinal changes of retinal thickness in mice during aging in vivo (Figure 2A). The OCT analysis showed no significant changes in the thickness of whole retina among different ages of mice (*p* = 0.957; Figure 2B). Similarly, the thickness of the GCC layer in the 4- (84.20 ± 2.98 μm, *p* = 0.318), 6- (83.88 ± 3.72 μm, *p* = 0.181), and 8-month-old mice (83.99 ± 3.93 μm, *p* = 0.214) showed no significant differences as compared to that in the 2-month-old mice (86.02 ± 2.22 μm; Figure 2C). In contrast, the thicknesses of the GCC layer in the 10- (81.29 ± 3.05 μm, *p* = 0.009), 12- (80.60 ± 4.19 μm, *p* = 0.001), 14- (80.70 ± 4.46 μm, *p* = 0.002), 16- (77.43 ± 4.30 μm, *p* < 0.001), and 18-month-old mice (77.47 ± 4.07 μm, *p* < 0.001) were significantly and progressively decreased by 5.50%, 6.30%, 6.18%, 9.99%, and 9.94% respectively as compared to that in the 2-month-old mice. Our results indicated progressive reduction of the thickness of the GCC layer in mouse retinas with increasing ages, starting from 10 months of age.

### 2.3. Changes of Retinal Ganglion Cell Density in Mice During Aging

**Figure 3:**
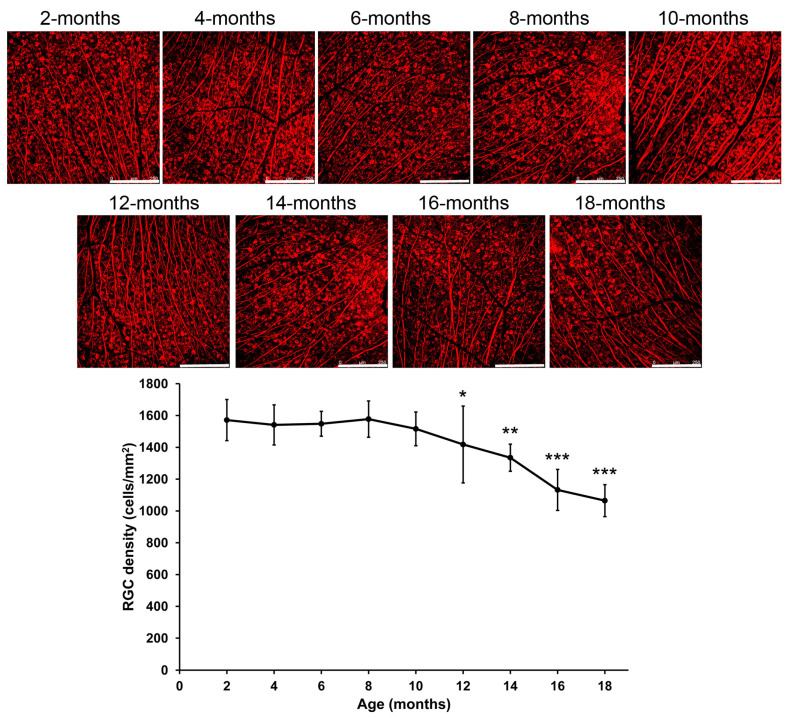
Changes of retinal ganglion cell density in mice during aging. Immunofluorescence analysis on the numbers of retinal ganglion cells (RGCs) in the retina of the 2- to 18-month-old mice. Scale bar: 200 μm. Red: β-III tubulin signal. Data were presented as mean ± standard deviation and compared by one-way analysis of variance with post hoc LSD test. * *p* < 0.05; ** *p* < 0.01; *** *p* < 0.001 as compared to the 2-month-old mice.

To delineate the progressive reduction in the thickness of the GCC layer, we further evaluated the RGC density by the immunofluorescence analysis. We found that there were no significant changes in the RGC density of RGCs in mice in 4- (1540.97 ± 125.99 cells/mm^2^, *p* = 0.722), 6- (1548.05 ± 78.19 cells/mm^2^, *p* = 0.759), 8- (1577.28 ± 114.13 cells/mm^2^, *p* = 0.937), and 10-month-old mice (1516.02 ± 106.04 cells/mm^2^, *p* = 0.489) as compared to that of the 2-month-old mice (1571.28 ± 129.23 cells/mm^2^; Figure 3). Instead, the densities of RGCs were significantly and progressively reduced in the 12- (1417.80 ± 241.68 cells/mm^2^, *p* = 0.039), 14- (1334.69 ± 85.20 cells/mm^2^, *p* = 0.003), 16- (1132.25 ± 128.97 cells/mm^2^, *p* < 0.001), and 18-month-old mice (1064.81 ± 100.35 cells/mm^2^, *p* < 0.001) by 9.77%, 15.06%, 27.94%, and 32.23% respectively as compared to that in the 2-month-old mice. Our results indicated the progressive RGC loss in mice with increasing ages, starting from 12 months of age.

### 2.4. Changes of Retinal Ganglion Cell Dendrites in Mice During Aging

**Figure 4:**
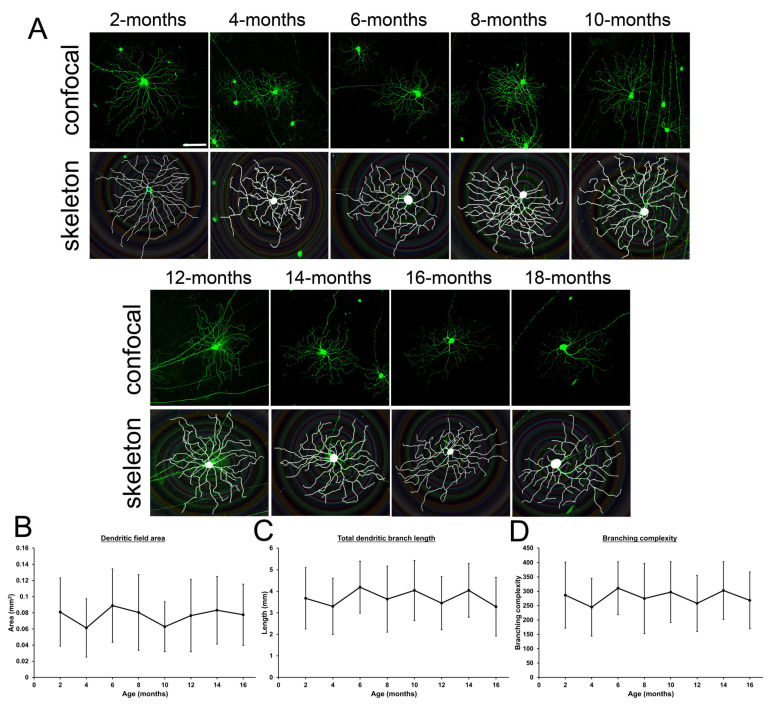
Changes of retinal ganglion cell dendrites in mice during aging. (**A**) Confocal microscopy images and dendritic skeleton images of retinal ganglion cell (RGC) dendrites of the 2- to 16-month-old Thy1-YFP-H mice. Scale bar: 200 μm. Green: yellow fluorescence protein signals. (**B**–**D**) Quantitative analysis on (**B**) the area of dendrite field, (**C**) total dendritic branch length, and (**D**) branching complexity, comparing to that of the 2-month-old mice. Data were presented as mean ± standard deviation and compared by one-way analysis of variance with post hoc LSD test.

Another potential influence on the GCC layer could be the dendritic shrinkage in RGCs. The dendritic morphology of RGCs was imaged in the retinal flatmount of the Thy1-YFP-H mice with different ages (Figure 4A). We found that the area of the dendritic field (0.081 ± 0.042 mm^2^ at 2-months to 0.078 ± 0.038 mm^2^ at 16-months, *p* > 0.05; Figure 4B), total length of dendritic branches (3.67 ± 1.42 mm at 2-months to 3.28 ± 1.36 mm at 16-months, *p* > 0.05; Figure 4C), and branching complexity (286.6 ± 115.0 at 2-months to 268.3 ± 98.9 at 16-months, *p* > 0.05; Figure 4D) showed no significant changes among different ages of mice. Our results indicated no significant changes of dendritic morphology in RGCs with increasing ages.

### 2.5. Changes of Cell Death Marker Expression in Mice During Aging

**Figure 5:**
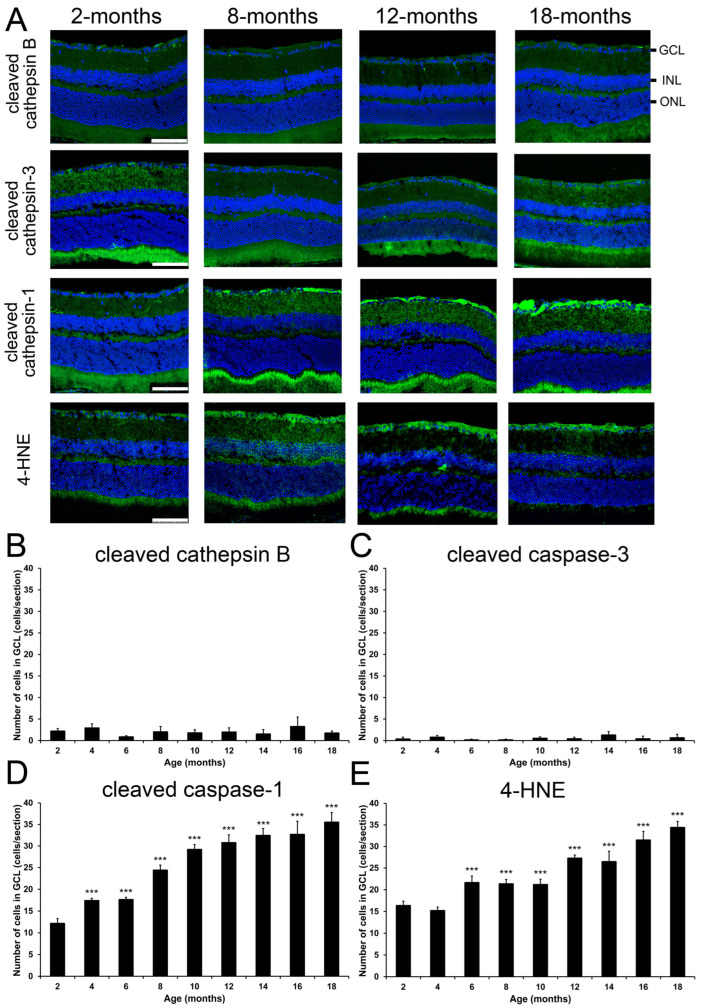
Changes of cell death marker expression in mice during aging. (**A**) Images of major time points (2, 8, 12, and 18-months) of the immunofluorescence analysis on cleaved cathepsin B (autolysis), cleaved caspase-3 (apoptosis), cleaved caspase-1 (pyroptosis), and 4-hydroxynonenal (4-HNE; ferroptosis) protein in the retinal sections. Scale bar: 100 μm. Green: Target antibody signal; Blue: DAPI nuclei counter-stain; GCL: ganglion cell layer; INL: inner nuclear layer; ONL: outer nuclear layer. (**B**–**E**) Quantification of (**B**) cleaved cathepsin B-, (**C**) cleaved caspase-3-, (**D**) cleaved caspase-1-, and (**E**) 4-HNE-positive stained cells in GCL of the 2- to 18-month-old mice. Data were presented as mean ± standard deviation and compared by one-way analysis of variance with post hoc LSD test. *** *p* < 0.001 as compared to the 2-month-old mice.

To delineate the RGC loss with increasing ages, we examined the 4 major modes of cells death (apoptosis, autolysis, pyroptosis, and ferroptosis) activated in RGCs post-optic nerve injury and reduced after senolytics treatment [13,14]. Immunofluorescence analysis demonstrated no significant change in the numbers of cleaved cathepsin B-positive cells (autolysis marker; 2.17 ± 0.62 cells/section at 2-months to 1.73 ± 0.49 cells/section at 18-months, *p* > 0.05; Figure 5A,B and Figure S2) and cleaved caspase-3-positive cells (apoptosis marker; 0.33 ± 0.47 cells/section at 2-months to 0.63 ± 0.82 cells/section at 18-months, *p* > 0.05; Figure 5A,C and Figure S2) in GCL among different ages of mice. Instead, the numbers of cleaved caspase-1-positive cells (pyroptosis marker) in GCL were significantly and progressively increased from the 4- (17.39 ± 0.55 cells/section, *p* < 0.001) to 18-month-old mice (35.50 ± 2.27 cells/section, *p* < 0.001) as compared to the 2-month-old mice (12.13 ± 1.14 cells/section; Figure 5A,D and Figure S2). Moreover, the number of 4-HNE-postive cells (ferroptosis marker) in GCL were also significantly and progressively increased from the 6- (21.63 ± 1.56 cells/section, *p* < 0.001) to 18-month-old mice (34.38 ± 1.43 cells/section, *p* < 0.001) as compared to the 2-month-old mice (16.33 ± 1.03 cells/section; Figure 5A,E and Figure S2). Our results indicated that pyroptosis and ferroptosis could be involved in the regulation of RGC loss as aging processes.

### 2.6. Changes of Cellular Senescence-Related Protein Expression in Mice During Aging

**Figure 6:**
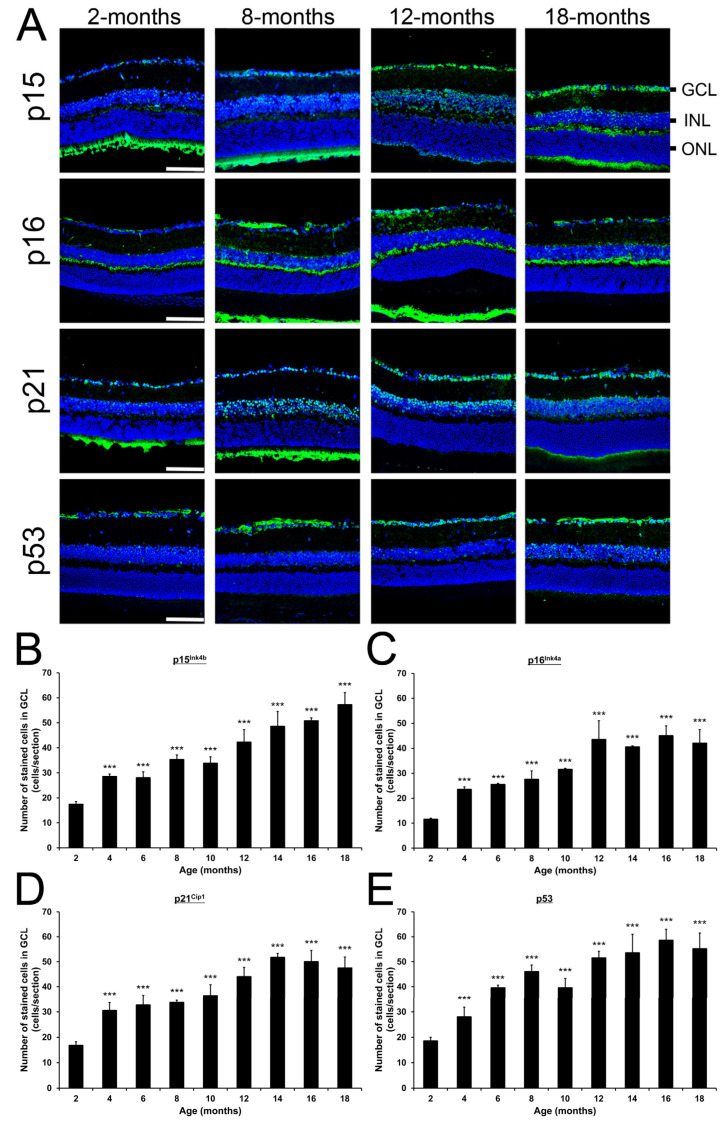
Changes of cellular senescence-related protein expression in mice during aging. (**A**) Images of major time points (2, 8, 12, and 18-months) of the immunofluorescence analysis on p15^Ink4b^, p16^Ink4a^, p21^Cip1^, and p53 protein in the retinal sections. Scale bar: 75 μm. Green: target antibody signal; Blue: DAPI nuclei counter-stain; GCL: ganglion cell layer; INL: inner nuclear layer; ONL: outer nuclear layer. (**B**–**E**) Quantification of (**B**) p15^Ink4b^-, (**C**) p16^Ink4a^-, (**D**) p21^Cip1^-, and (**E**) p53-positively stained cells in GCL of the 2- to 18-month-old mice. Data were presented as mean ± standard deviation and compared by one-way analysis of variance with post hoc LSD test. *** *p* < 0.001 as compared to the 2-month-old mice.

To further evaluate the involvement of cellular senescence with increasing ages, we determined the expression of cellular senescence-related proteins (p15^Ink4b^, p16^Ink4a^, p21^Cip1^, and p53) in retina among different ages of mice. Immunofluorescence analysis demonstrated p15^Ink4b^, p16^Ink4a^, p21^Cip1^, and p53 are all expressed in GCL among mice of different ages (Figure 6A and Figure S3). The numbers of p15^Ink4b^-positive cells in GCL were significantly and progressively increased from the 4- (28.50 ± 1.00 cells/section, *p* < 0.001) to 18-month-old mice (57.17 ± 4.92 cells/section, *p* < 0.001) as compared to the 2-month-old mice (17.40 ± 1.14 cells/section; Figure 6B). The numbers of p16^Ink4a^-positive cells in GCL were also significantly and progressively increased from the 4- (23.50 ± 1.12 cells/section, *p* < 0.001) to 12-month-old mice (43.50 ± 7.50 cells/section, *p* < 0.001) as compared to the 2-month-old mice (11.50 ± 0.50 cells/section), and reached a plateau from 12-months to 18-months (42.00 ± 5.43 cells/section, *p* < 0.001; Figure 6C). Similarly, the numbers of p21^Cip1^-positive cells were significantly and progressively increased from the 4- (30.50 ± 3.20 cells/section, *p* < 0.001) to 14-month-old mice (51.67 ± 1.70 cells/section, *p* < 0.001) as compared to the 2-month-old mice (16.75 ± 1.48 cells/section), and reached a plateau from 14-months to 18-months (47.43 ± 4.47 cells/section, *p* < 0.001; Figure 6D). Moreover, the numbers of p53-positive cells in GCL were significantly and progressively increased from the 4- (28.00 ± 3.87 cells/section, *p* < 0.001) to 16-month-old mice (58.50 ± 4.56 cells/section, *p* < 0.001) as compared to the 2-month-old mice (18.50 ± 1.50 cells/section), and reached a plateau from 16-months to 18-months (55.17 ± 6.31 cells/section, *p* < 0.001; Figure 6E). Our results indicated that the cellular senescence-related cell cycle arrest proteins could be involved in the regulation of cellular senescence with increasing ages.

### 2.7. Retinal Transcriptomic Analysis

**Figure 7:**
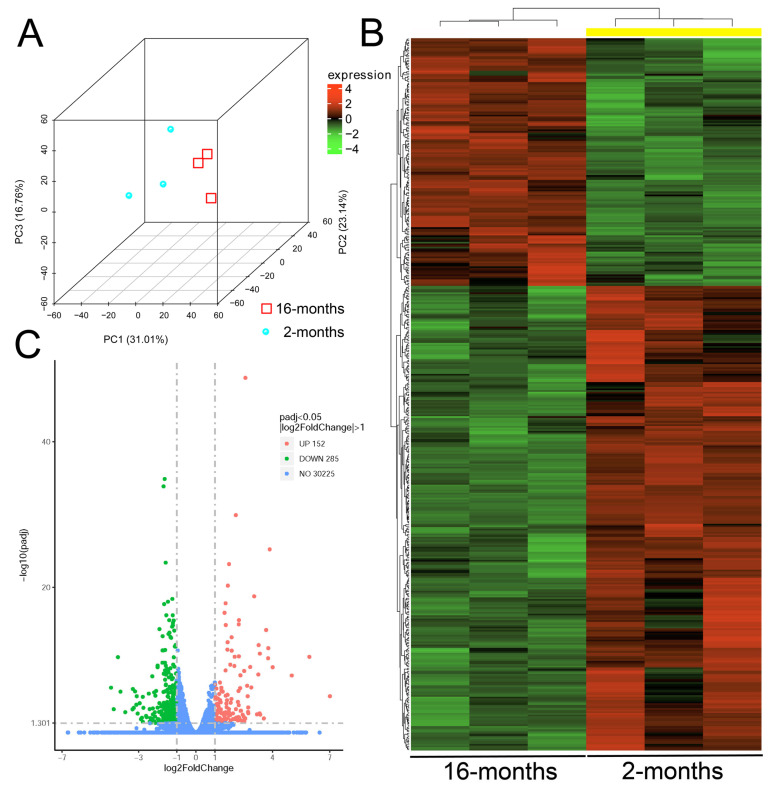
RNA sequencing analysis on the retina of the 2- and 16-month-old mice. (**A**) Principle component analysis on the whole transcriptome profiles of the retinas of the 2- (blue dots) and 16-month old mice (red squares). (**B**) Hierarchical clustering analysis of differentially expressed genes in the retinas of the 2- and 16-month old mice. (**C**) Volcano plots of gene expression changes in the retina of the 16-month-old mice as compared to that of the 2-month-old mice by the independent T-test. Red dots: Significantly upregulated genes; Green dots: Significantly downregulated genes; Blue dots: No significant changes. Significant differential expression was defined as log_2_ fold change ≥ 1 and corrected *p* < 0.05.

To further delineate the additional mechanisms for aging, we conducted RNA sequencing analysis on mouse retinas of different ages. Principle component analysis (Figure 7A) and hierarchical clustering analysis (Figure 7B) confirmed the differential clustering of the gene expression profiles between the retinas of 2- and 16-months old mice. Of total 30,662 genes identified in mouse retina, 437 genes showed differential expression in the retina of 16-month-old mice as compared to that of the 2-month-old mice, including 152 upregulated and 285 downregulated genes (Figure 7C and Table S2). Functional annotation clustering in gene ontology analysis showed that the differentially expressed genes were involved in the pathways of neurodegeneration (enrichment score = 4.21, *p* = 4.13 × 10^−4^), innate immunity (enrichment score = 2.89, *p* = 5.49 × 10^−5^), mitochondrial ATP synthesis (enrichment score = 2.76, *p* = 2.81 × 10^−5^), PI3K-Akt signaling pathway (enrichment score = 1.71, *p* = 5.13 × 10^−4^), synapse disassembly (enrichment score = 1.40, *p* = 9.18 × 10^−5^), positive regulation of tumor necrosis factor production (enrichment score = 1.37, *p* = 0.016), and retina development (enrichment score = 1.31, *p* = 0.043) (Table 1).

**Figure 8:**
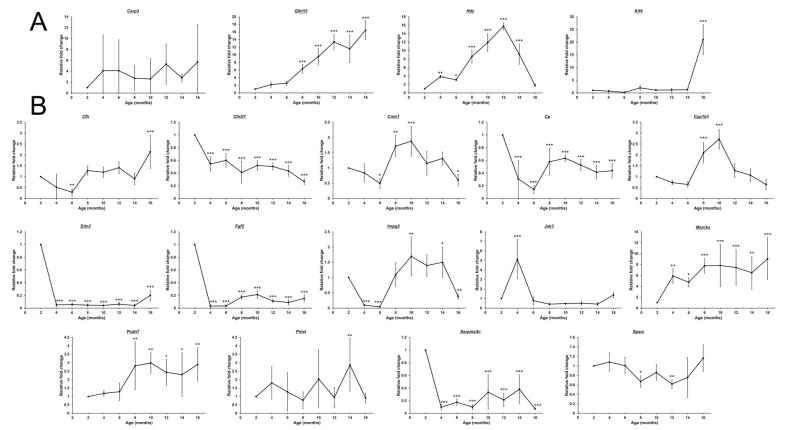
Gene expression analysis on the retinas of mice during aging. SYBR green polymerase chain reaction on the expression of significantly (**A**) upregulated (*Csrp3*, *Glb1l3*, *Hdc*, and *Kif4*) and (**B**) downregulated genes (*Cfh*, *Chi3l1*, *Cntn1*, *Cp*, *Cyp1b1*, *Edn2*, *Fgf2*, *Impg2*, *Jak3*, *Marcks*, *Pcdh7*, *Pmel*, *Serpina3n*, and *Sparc*) identified by the RNA sequencing analysis in the retina of 2- to 16-month-old mice, comparing to that of the 2-month-old mice. *Actb* was used as housekeeping gene for normalization. Data presented as mean of relative fold change (2^−ΔΔCt^) ± standard deviation and compared by one-way analysis of variance with post hoc LSD test. * *p* < 0.05; ** *p* < 0.01; *** *p* < 0.001.

To confirm the results from the RNA sequencing analysis, 18 differentially expressed genes were selected based on their expression and relationship with glaucoma, RGCs, and inflammation, and verified in mouse retinas of different ages (2–16 months) by SYBR Green PCR analysis. For the upregulated genes (*Csrp3*, *Glb1l3*, *Hdc*, and *Kif4*) identified by the RNA sequencing analysis, the expressions of *Csrp3* and *Hdc* by SYBR Green PCR analysis did not follow the trend as the RNA sequencing analysis in the 16-month-old mice (*p* > 0.05); yet, *Hdc* were significantly upregulated in the retinas from 4- (3.84 ± 0.33 folds, *p* = 0.001) to 14-month-old mice (9.13 ± 2.37 folds, *p* < 0.001) with a peak at the 12-month-old mice (15.75 ± 0.84 folds, *p* < 0.001) as compared to that of the 2-month-old mice (Figure 8A). The expression of *Kif4* was significantly upregulated only in the retinas of 16-month-old mice (21.03 ± 5.96 folds, *p* < 0.001), while the expression of *Glb1l3* was significantly and progressively upregulated in the retinas from 8- (6.33 ± 1.17 folds, *p* < 0.001) to 16-month-old mice (16.44 ± 2.45 folds, *p* < 0.001). For the downregulated genes (*Cfh*, *Chi3l1*, *Cntn1*, *Cp*, *Cyp1b1*, *Edn2*, *Fgf2*, *Impg2*, *Jak3*, *Marcks*, *Pcdh7*, *Pmel*, *Serpina3n*, and *Sparc*) identified by the RNA sequencing analysis, the expressions of *Cfh*, *Cyp1b1*, *Jak3*, *Marcks*, *Pcdh7*, *Pmel*, and *Sparc* by SYBR Green PCR analysis did not follow the respective trend as the RNA sequencing analysis in the 16-month-old mice (*p* > 0.05; Figure 8B). The expressions of *Cntn1* (0.60 ± 0.19 folds, *p* = 0.044) and *Impg2* (0.38 ± 0.09 folds, *p* = 0.008) were significantly downregulated in the retinas of 16-month-old mice as compared to that of the 2-month-old mice, while the expressions of *Cp* (0.31 ± 0.29 folds, *p* < 0.001 to 0.44 ± 0.11 folds, *p* < 0.001), *Edn2* (0.05 ± 0.03 folds, *p* < 0.001 to 0.20 ± 0.09 folds, *p* < 0.001), *Fgf2* (0.03 ± 0.02 folds, *p* < 0.001 to 0.15 ± 0.06 folds, *p* < 0.001), and *Serpina3n* (0.09 ± 0.05 folds, *p* < 0.001 to 0.07 ± 0.01 folds, *p* < 0.001) were significantly and steadily in the retinas from 4- to 16-month-old mice. Only *Chi3l1* showed significantly and progressively downregulated expression in the retinas from 4- (0.54 ± 0.11 folds, *p* < 0.001) to 16-month-old mice (0.27 ± 0.05 folds, *p* < 0.001). Our results suggested that the neurodegeneration pathways, inflammation, and mitochondrial ATP synthesis as well as the progressive differential expression of *Glb1l3* and *Chi3l1* genes could be related to the aging process in mouse retina.

## 3. Discussion

Results from this study showed: (1) Progressive elevation of senescent cells and cellular senescence-related cell cycle arrest proteins in mouse retinas with increasing ages; (2) Progressive RGC loss and reduction of the thickness of the GCC layer in mouse retinas with increasing ages; (3) No significant change in the dendritic morphology of RGCs with increasing ages; (4) Progressive elevation of pyroptosis and ferroptosis markers in GCL with increasing ages; (5) The differentially expressed genes related to the neurodegeneration pathways, innate immunity, and mitochondrial ATP synthesis involved in the aging process in mouse retina. Collectively, this study demonstrated the association of cellular senescence with RGC loss in mice with increasing ages.

The numbers of RGCs on aging studies have been assessed by different RGC markers in different species. It has been reported no RGC loss in the comparison of the 5-month-old with 20–21-month-old C57BL/6 mice by the Brn3a immunofluorescence analysis [10]. Similarly, no significant differences were found between the post-natal day (P) 60 and P365 Sprague Dawley (SD) rats analyzed by Fluorogold labeling and Brn3a immunofluorescence analysis [12], and from 3-month-old to 30-month-old SD rats by the cresyl violet staining analysis [11]. Moreover, the RGC cell densities also showed no significant differences between 2–6-month-old and 11–15-month-old marmosets by the RNA binding protein with multiple splicing (RBPMS) immunofluorescence analysis [16]. In contrast, approximately 46% of RGC loss was reported in 18-month-old C57/BL6 mice as compared to the 3-month-old mice by Fluorogold labeling [7], and the RGC density of 20-month-old C57BL/6J mice was found to be lower than that of the 3-month-old mice by the RBPMS immunofluorescence analysis [8]. Besides, the numbers of RGCs in the retinal section were found to be significantly lower in the 12-, 18-, and 24-month-old Albino Wistar rats as compared to the 5-month-old rats by the Brn3a immunofluorescence analysis [9]. In this study, we observed significant reduction of GCC layer thickness and RGC loss beginning in the 10- and 12-month-old mice respectively (Figure 2 and Figure 3), which corresponds to a human age in the forties (according to the white paper from Jackson Laboratory; https://resources.jax.org/white-papers/whitepaper-aged-b6, accessed on 16 March 2024), the age of adult-onset primary open angle glaucoma begins. Further progressive reduction of GCC layer thickness (9.94%) and RGC loss (32.23%) can be found in the 18-month-old mice (corresponds to a human age in the sixties). Therefore, our results indicated 0.62%/months (9.94%/(18−2) months) of age-related GCC layer thinning (roughly 0.25%/year in human (9.94%/(60-20 years))) and 2.01%/months (32.23%/(18−2) months) of age-related RGC loss (roughly 0.81%/year in human (32.23%/(60-20 years))) from 2-months old mice (corresponding to a human age in the twenties) to 18-months old mice, which is close to a previous report of 0.27%/year of age-related RNFL thinning and 0.50%/year of age-related RGC loss in human [6]. The receptor of the dopaminergic system, by modulating the IOP, could be involved in the RGC survival regulation in glaucoma [17].

At present, research studies investigating the dendritic morphology of RGCs in animals with increasing ages are limited. An earlier study reported that the diameter of RGC dendritic arbors and the dendritic area of RGCs in Thy1-YFP-H mice decrease with age [18]. In contrast, a recent study showed that there were no obvious differences in the dendritic morphology of RGCs between 3- and 12-month-old ON- and OFF-RGCs of Thy1-YFP-H mice [19]. In this study, we also did not observe statistically significant changes in the area of the dendritic field, total length of dendritic branches, and branching complexity among different ages of Thy1-YFP-H mice (Figure 4). Instead of the natural morphology at different ages, the RGC dendrites could have differential susceptibilities to IOP elevation in young and old mice [19]. Further investigations can evaluate the dendritic morphology in different ages of mice upon stimulation with different pathophysiological factors.

Age-related molecular changes can provide insights into age-related disease biology. Human plasma proteome profiles discovered non-linear undulating waves of changes in aging, and the differentially expressed proteins are peaks at the age of 34, 60, and 78 [20]. Consistently, in this study, we found that the number of SA-βgal-positive cells began significantly increasing in 8-months old mice (Figure 1), which corresponds to human age of thirties. Apart from the increased lysosomal mass reflected by the SA-βgal activity, cell cycle arrest is another hallmark of cellular senescence, which can be reflected by the upregulation of p15^Ink4b^, p16^Ink4a^, p21^Cip1^, and p53 [21,22]. It has been reported that p53 regulates RGC death induced by N-methyl-D-aspartate (NMDA) in mice [23], and overexpression of p15^Ink4b^ can further reduce RGC viability in NMDA-injected mouse retinas [24]. Moreover, upregulation of p16^Ink4a^ has been shown to induce RGC senescence in ischemic injury mouse model [25], while p21^Cip1^ expression is strongly correlated with the SA-βgal staining in the developing avian retina [26]. In this study, we observed that the cell cycle arrest-related proteins p15^Ink4b^, p16^Ink4a^, p21^Cip1^, and p53 were all expressed in GCL, and their expressions were significantly increased in mice with increasing ages (Figure 6), further indicating the increase in cellular senescence in mice with aging. In addition, another hallmark of cellular senescence is the senescence-associated secretory phenotype (SASP) [22], and inflammation is a core in SASP [15]. In this study, we found that the clusters of the differentially expressed genes from the RNA sequencing analysis (Figure 7) are involved in innate immunity and positive regulation of tumor necrosis factor production (Table 1). It has been reported that innate immune sensor controls the SASP [27], suggesting that the activation of the SASP and increase in cellular senescence as aging processes. Furthermore, pyroptosis is a pro-inflammatory and lytic form of cell death [28]. In this study, we observed a significant and progressive increase in the expression of pyroptosis marker in GCL of mice between 4 and 18 months of age (Figure 5 and Figure S2), supporting the presence of heightened inflammation during aging [29]. Collectively, our results suggest that cellular senescence may contribute to RGC loss in aging mice. Together with the involvement of cellular senescence in RGC survival regulation in the optic nerve injury model [14], RGC death triggered by cellular senescence could be a general phenomenon in physiological and pathological conditions. Early removal of senescent cells could potentially prevent further cell loss to maintain adequate number of functional cells for essential tissue functions [30].

There were several limitations in this study. First, whole retina was used in the RNA sequencing and gene expression analyses. Future studies are needed to investigate the biological roles of the differentially expressed genes specifically in RGCs during aging and upon injury so as to delineate the specific related mechanisms. Second, we assessed the expression of cellular senescence and cell death markers in GCL by immunofluorescence analysis in retinal sections. Co-staining of the markers with RGC marker can help to confirm their expressions in RGCs. Besides, older mice (>18-month-old) were not investigated in this study although clear trends of RGC loss and increased cellular senescence have already been found in mice older than 12-month-old. Future investigations on older mice can help to confirm the findings in this study.

## 4. Materials and Methods

### 4.1. Animals

Male C57BL/6J (Beijing Vital River Laboratory Animal Technology Co. Ltd., Beijing, China) and B6.Cg-Tg(Thy1-YFP)HJrs/J (Thy1-YFP-H) mice (Jackson Laboratory, Bar Harbor, ME; for dendrite analyses) of different ages (2–18-month-old) were maintained in a specific pathogen-free grade animal facility at 22 ± 1 °C and 40 ± 10% humidity with 12-h dark/light cycle. Standard rodent chow and water were provided *ad libitum*. The experimental protocols were approved by the Animal Experimentation Ethics Committee of the Joint Shantou International Eye Center of Shantou University and the Chinese University of Hong Kong (approval number: EC20220830(6)-P03) and executed according to the Guidelines of the Association for Research in Vision and Ophthalmology Statement on Use of Animals in Ophthalmic and Vision Research. Three mice were used in each group for the RNA sequencing analysis, and five mice were used in each group for other experiments.

### 4.2. Senescence-Associated Β-Galactosidase Staining

The senescence-associated β-galactosidase (SA-βgal) staining were performed with the Senescence β-Galactosidase Staining Kit (Cell Signaling Technology). Briefly, the C57BL/6J mice of different ages (2–18-month-old) were sacrificed and perfused with 4% paraformaldehyde (Sigma-Aldrich, St. Louis, MO, USA). The eyeballs were enucleated for post-fixation in 4% paraformaldehyde at 4 °C for overnight. The fixed eyeballs were cryoprotected with 10–30% sucrose gradient and cryo-embedded in optimal cutting temperature compound. The cryo-sections (10 μm) with the pupil-ON position were blocked with the Fixative Solution at room temperature for 20 min, followed by incubating with the β-Galactosidase Staining Solution (pH 6.0) at 37 °C for 48 h [25]. The stained sections were mounted with 70% glycerol, and the blue signals were visualized under a light microscopy (Leica DM4B, Leica Microsystems, Wetzlar, Germany). For each group, 20 retinal images at 200 μm from the ON were analyzed.

### 4.3. Ganglion Cell Complex Layer Analysis

Cross-sectional retina of the mice was imaged by the spectral domain-optical coherence tomography (OCT) mode of the RETImap^®^ machine (Roland Consult, Brandenburg an der Havel, Germany) in vivo. Briefly, the C57BL/6J mice of different ages (2–18-month-old) were anesthetized with intramuscular injection of a mixture (0.1 mL/100 g) of 70 mg/mL Zoletil^®^ (Virbac) and 20 mg/mL xylazine (Sigma-Aldrich). Retinal images were taken from the mice given with a 100-diopter corneal contact lens. Two cross-sectional images were taken across the optic nerve head (as reference point) for each eye of the mice. The thickness of the ganglion cell complex (GCC), which is composed of RNFL, ganglion cell layer (GCL), and inner plexiform layer, was measured by the ImageJ software at 300 μm from the optic papilla at the nasal, temporal, superior, and inferior positions.

### 4.4. Retinal Ganglion Cell Survival Analysis

The numbers of RGCs in the retina was evaluated by the immunofluorescence analysis according to our previous protocol [31]. Briefly, the C57BL/6J mice of different ages (2–18-month-old) were sacrificed and perfused with 4% paraformaldehyde. The eyeballs were enucleated, and the retinas were dissected and further fixed with 4% paraformaldehyde for 2 h. After fixation, the retinas were blocked and permeabilized with 5% normal goat serum (NGS) and 0.3% Triton X-100 in phosphate buffered saline (PBS) at room temperature for 1 h, and probed with the anti-neuron-specific βIII-tubulin antibody (catalog number: 5568; Cell Signaling Technology, Danvers, MA, USA) at 4 °C overnight, followed by incubating with the Alexa Fluor-555-conjugated secondary antibody (Thermo Fisher Scientific, Waltham, MA, USA) at room temperature for 2 h. The stained retinas were mounted with anti-fading mounting medium and imaged in 8 specified fields for each retina (Supplementary Figure S4; each field: 0.788 × 0.788 mm^2^) under a confocal microscope (Leica TCS SP5 II, Leica Microsystems, Wetzlar, Germany) according to our previous study [32]. The numbers of the positively stained cells were counted manually using the Point Tool in the ImageJ software (version 1.47; National Institutes of Health, Bethesda, MD, USA). The density of positively stained cells per field was calculated with the division by the retinal area, and mean density of RGCs in each retina was determined.

### 4.5. Retinal Ganglion Cell Dendrite Analysis

The RGC dendrite analysis was performed according to our previous study [32]. Briefly, the Thy1-YFP-H mice of different ages (2–16-month-old) were sacrificed and perfused with 4% paraformaldehyde. The eyes were enucleated and post-fixed with 4% paraformaldehyde for 2 h. The retinas were dissected and flatmounted on a glass slide. Individual RGC was imaged by a confocal microscope (Leica TCS SP5 II). The dendrite analysis was performed using the Fiji software (version Latest) [33] with the Simple Neurite Tracer [34] and Sholl Analysis plug-ins [35]. The area of dendritic field (the area bounded by connected line segments joining the ends of all terminal dendritic branches), total dendritic branch length (the sum of the lengths of all dendrites), and dendritic branching complexity (total number of intersections between RGC filament and concentric circles drawn at 10-μm intervals from the soma using Sholl analysis) were analyzed in each imaged RGC. For each mouse, at least 20 RGCs were analyzed.

### 4.6. Immunofluorescence Analysis

The C57BL/6J mice of different ages (2–18-month-old) were sacrificed and perfused with 4% paraformaldehyde. The eyeballs were enucleated for post-fixation in 4% paraformaldehyde at 4 °C for overnight. The fixed eyeballs were cryo-protected with 10–30% sucrose gradient and cryo-embedded in optimal cutting temperature compound (Leica). The cryo-sections (10 μm) with pupil-ON position were blocked and permeabilized with 5% NGS and 0.3% Triton X-100 in PBS at room temperature for 1 hr, and probed with the primary antibodies against p15^Ink4b^ (catalog number: ab53034; Abcam, Cambridge, the U.K.), p16^Ink4a^ (catalog number: ab108349; Abcam), p21^Cip1^ (catalog number: ab188224; Abcam), p53 (catalog number: ab32049; Abcam), cleaved caspase-3 (catalog number: 9661; Cell Signaling Technology, Danvers, MA, USA), cleaved cathepsin B (catalog number: ab214428; Abcam), cleaved caspase-1 (catalog number: ab179515; Abcam), and 4-hydroxynonenal (4-HNE; catalog number: ab46545, Abcam) at 4 °C for overnight, followed by the incubation with respective secondary antibodies conjugated with Alexa Fluor-488 at room temperature for 2 hr. The stained sections were mounted with the anti-fading mounting medium, and the fluorescence signals were visualized by a confocal microscopy (Leica TCS SP5 II). For each group, 20 retinal images at 200 μm from the ON were analyzed, and the number of positively stained cells in the GCL was counted in each retinal section.

### 4.7. Retinal Transcriptomic Analysis

Retinal transcriptomic analysis was conducted according to our previous study [36]. The 2- and 16-month-old C57BL/6J mice were sacrificed, and the retinas were dissected. Total RNA of the retina was extracted and purified with the TRIzol reagent (Thermo Fisher Scientific) according to the manufacturer’s protocols. RNA sequencing experiments were conducted by the Novogene Co. Ltd. (Beijing, China). Briefly, the RNA integrity was evaluated by the Agilent Bioanalyzer 2100 (Agilent Technologies, Santa Clara, CA, USA) using the RNA Nano 6000 Assay Kit (Agilent Technologies). The mRNA was isolated from total RNA using oligo dT magnetic beads, and the complementary DNA was synthesized by the M-MuL V Reverse Transcriptase and DNA Polymerase I. With the adenylation at the 3’ ends of the DNA fragments, the adaptor with the hairpin loop structure was ligated for hybridization. Polymerase chain reaction (PCR) was performed on the 370–420-base pairs (bp) cDNA fragments purified by the AMPure XP kit (Beckman Coulter Life Sciences, Indianapolis, IN, USA) with the Phusion High-Fidelity DNA polymerase, universal PCR primers, and index (X) primer. The PCR products were purified, and the library quality was assessed by the Agilent Bioanalyzer 2100. The library preparations were sequenced using the Illumina Novaseq 6000 platform to generate 150-bp paired-end reads. After removing the low-quality reads and reads containing the adapter or poly-N from raw data, the paired-end clean reads were aligned to the reference genome using Hisat2 v2.0.5. FeatureCounts v1.5.0-p3 was used to count the number of reads mapped to each gene, and the fragments per kilobase of transcript sequence per millions base pairs sequenced (FPKM) of each gene was calculated. Principle component analysis (PCA) based on the whole transcriptome profiles was adopted to confirm the distinct clustering of different groups. The volcano plot was used to present the gene expression changes in the retinas of the 16-months old mice as compared to that of the 2-months old mice. Differential gene expression analysis was performed using the DESeq2 R package (1.20.0). The *p*-values were adjusted using the Benjamini & Hochberg method to control the false discovery rate. Differential gene expression was considered as log_2_ fold change ≥1 or ≤−1 and corrected *p* (*P_corr_*) < 0.05. Hierarchical clustering analysis of differentially expressed genes was also used to visualize the distinct gene expression patterns in different groups.

Gene ontology analysis of the differential expressed genes was evaluated by DAVID Bioinformatics Resources 6.8 [37]. Enrichment score > 1.3 was considered as statistically significant.

Based on the gene ontology analysis and expressions, 18 differentially expressed genes were selected for further SYBR green PCR validation in mice of different ages (2–16-month-old). Total RNA of the mouse retina was converted to complementary DNA using the SuperScript III reverse transcriptase (Thermo Fisher Scientific) and amplified by SYBR Green I Master Mix (TaKaRa Bio Inc., Shiga, Japan) in the LightCycler 480 system (Roche, Basel, Switzerland) with respective specific primers (Supplementary Table S1). *Actb* was used as the housekeeping gene for normalization. Relative expression was calculated by the 2^−ΔΔCt^ method as compared to the 2-months old mice.

### 4.8. Statistical Analysis

Data were presented as the mean of the results from 5 mice ± standard deviation (SD), and compared by one-way analysis of variance (ANOVA) with post-hoc Fisher’s least significant difference (LSD) test. All statistical analyses were performed using the IBM SPSS Statistics 26 (SPSS Inc., Chicago, IL, USA). *p* < 0.05 was considered as statistically significance.

## 5. Conclusions

This study revealed the increase in cellular senescence and progressive RGC loss in mice during aging, possibly mediated through pyroptosis and ferroptosis. Targeting cellular senescence and the related cell death pathways could potentially help to alleviate the age-related RGC reduction so as to maintain adequate number of RGCs for essential vision throughout life.

## Figures and Tables

**Table 1:** Gene ontology analysis on the differentially expressed genes in the retinas of 2- and 16-month-old mice.

Functional Annotation Clusters	Enrichment Score	Gene Count	%	*p*
Secreted	5.59	53	14.21	1.61 × 10^−7^
Glycoprotein	4.87	95	25.47	5.80 × 10^−6^
Pathways of neurodegeneration	4.21	19	5.09	4.13 × 10^−4^
Cell adhesion	4.01	29	7.77	4.36 × 10^−8^
Krueppel-associated box	3.82	21	5.63	1.01 × 10^−6^
Innate immunity	2.89	18	4.83	5.49 × 10^−5^
Mitochondrial ATP synthesis coupled proton transport	2.76	8	2.14	2.81 × 10^−5^
Plastocyanin-like	2.63	3	0.80	5.38 × 10^−4^
Extracellular matrix organization	2.57	13	3.49	1.25 × 10^−5^
Immunoglobulin subtype 2	1.83	11	2.95	0.002
Cytoskeleton	1.82	36	9.65	4.73 × 10^−4^
Proton-transporting ATP synthase complex, coupling factor F(o)	1.81	3	0.80	0.017
PI3K-Akt signaling pathway	1.71	16	4.29	5.13 × 10^−4^
Developmental protein	1.65	25	6.70	0.025
Metal ion binding	1.62	77	20.64	5.47 × 10^−4^
Leukocyte cell-cell adhesion	1.50	4	1.07	0.009
Glycoside hydrolase superfamily	1.49	4	1.07	0.044
ECM-receptor interaction	1.41	7	1.88	0.003
Synapse disassembly	1.40	4	1.07	9.18 × 10^−5^
Positive regulation of tumor necrosis factor production	1.37	7	1.88	0.016
Retina development	1.31	5	1.34	0.043

## Data Availability

The data can be made available with reasonable request to the corresponding author.

## Supplementary Materials

The following supporting information can be downloaded at: https://www.mdpi.com/article/10.3390/ijms26125436/s1, Figure S1: Enlarged image of senescence-associated β-galactosidase expressed cells in ganglion cell layer.; Figure S2: Cell death marker expression from 2-month-old to 18-month-old mice.; Figure S3: Cellular senescence-related protein expression from 2-month-old to 18-month-old mice; Figure S4: Illustration of the image position of retinal wholemount analysis on retinal ganglion cells; Table S1: Primers for gene expression analysis.; Table S2: Differentially expressed genes in the retina of the 16-month-old mice compared to that of the 2-month-old mice.
