# Supplementary material for: The Profile of Retinal Ganglion Cell Death and Cellular Senescence in Mice with Aging
Source: Int J Mol Sci. 2025 Jun 6;26(12):5436. doi: 10.3390/ijms26125436 (PMC12192622; doi:10.3390/ijms26125436)
Supplement: Supplementary file 1 [file ijms-26-05436-s001.zip › ijms-3619794-supplementary.pdf]

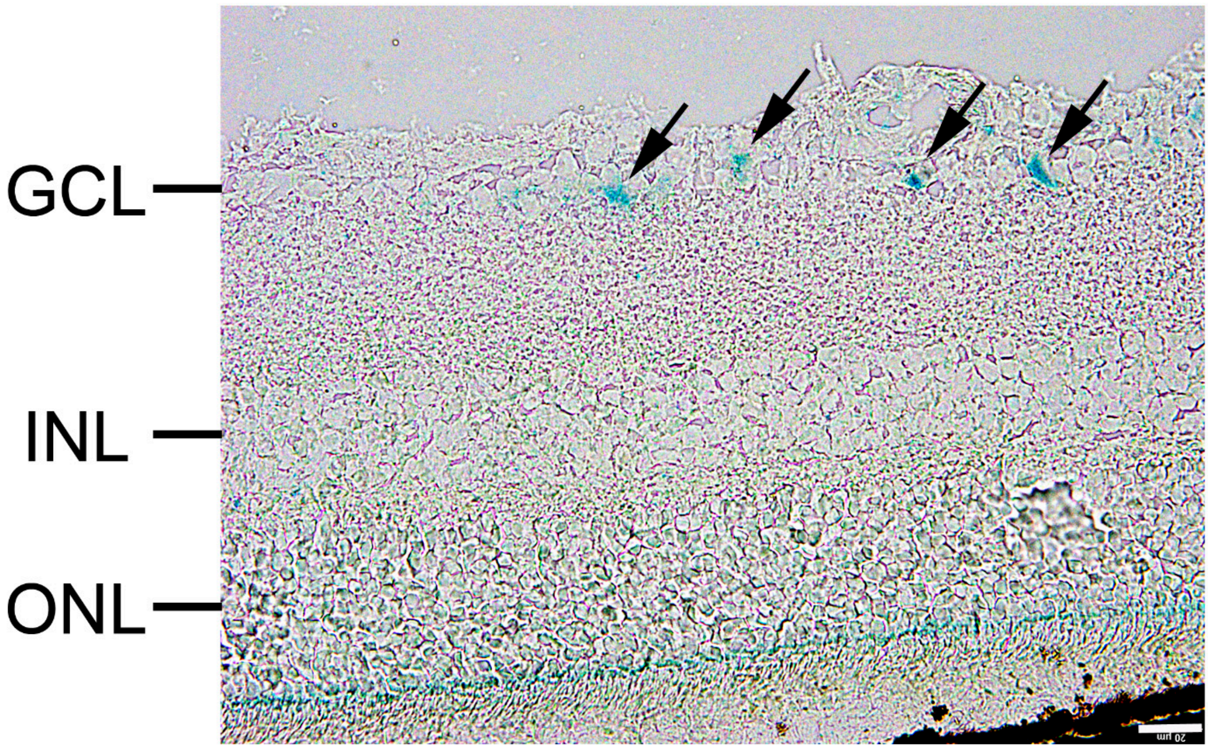

Supplementary figure S1: Enlarged image of senescence-associated β-galactosidase expressed cells in ganglion cell layer.

Arrow: positively stained cells in the ganglion cell layer (GCL); Blue: senescence-associated β-galactosidase (SA-βgal) activity. INL: inner nuclear layer; ONL: outer nuclear layer; Scale bar: 20 μm.

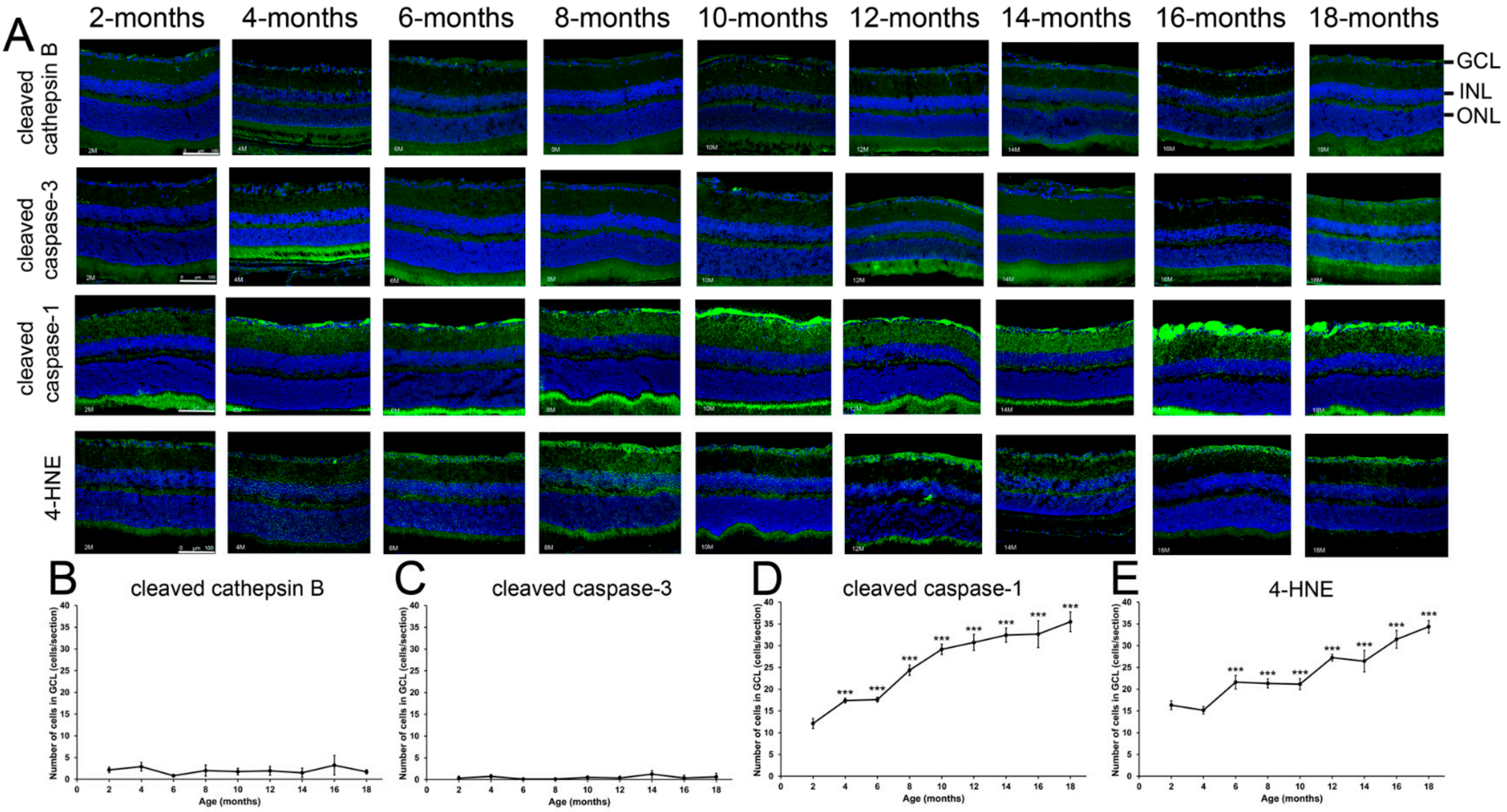

Supplementary figure S2: Cell death marker expression from 2-month-old to 18-month-old mice.

(**A**) Immunofluorescence analysis of (**B**) cleaved cathepsin B (autolysis), (**C**) cleaved caspase-3 (apoptosis), (**D**) cleaved caspase-1 (pyroptosis), and (**E**) 4-hydroxynonenal (4-HNE; ferroptosis) protein in the retina of the 2- to 18-month-old mice and (**B** – **E**) quantification in ganglion cell layer (GCL). Scale bar: 100 μm. Green: Target antibody signal; Blue: DAPI nuclei counter-stain. INL: inner nuclear layer; ONL: outer nuclear layer. Data were presented as mean ± standard deviation and compared by one-way analysis of variance with post hoc LSD test. $^{***}P < 0.001$ as compared to the 2-month-old mice.

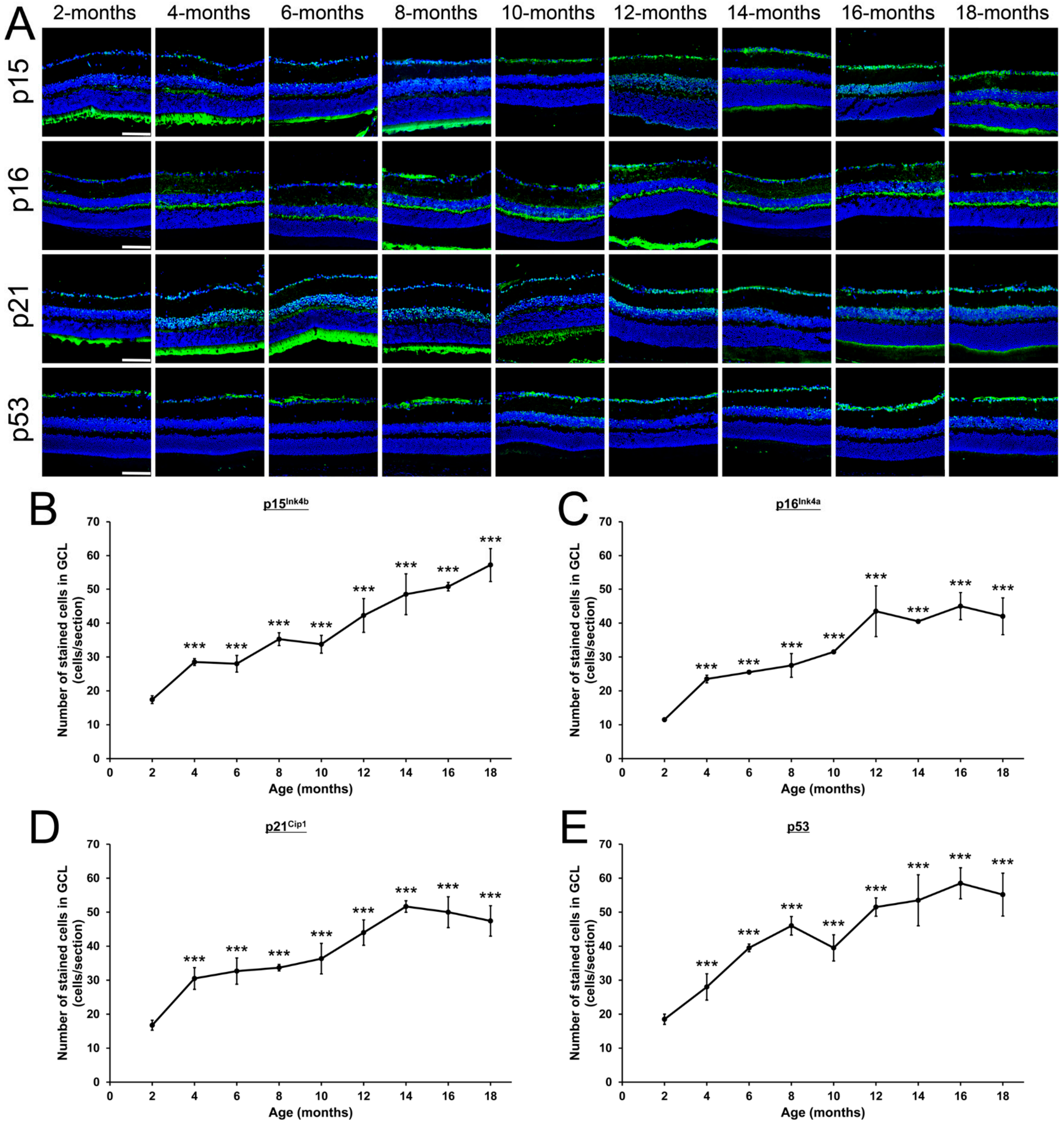

Supplementary figure S3: Cellular senescence-related protein expression from 2-month-old to 18-month-old mice.

(**A**) Immunofluorescence analysis of $p15^{Ink4b}$, $p16^{Ink4a}$, $p21^{Cip1}$, and p53 protein in the retina of the 2- to 18-month-old mice and (**B** – **E**) quantification of (**B**) $p15^{Ink4b}$, (**C**) $p16^{Ink4a}$, (**D**) $p21^{Cip1}$, and (**E**) p53 in ganglion cell layer (GCL). Scale bar: 75 μm. Green: target antibody signal; Blue: DAPI nuclei counter-stain. INL: inner nuclear layer; ONL: outer nuclear layer. Data were presented as mean ± standard deviation and compared by one-way analysis of variance with post hoc LSD test. $^{***}P < 0.001$ as compared to the 2-month-old mice.

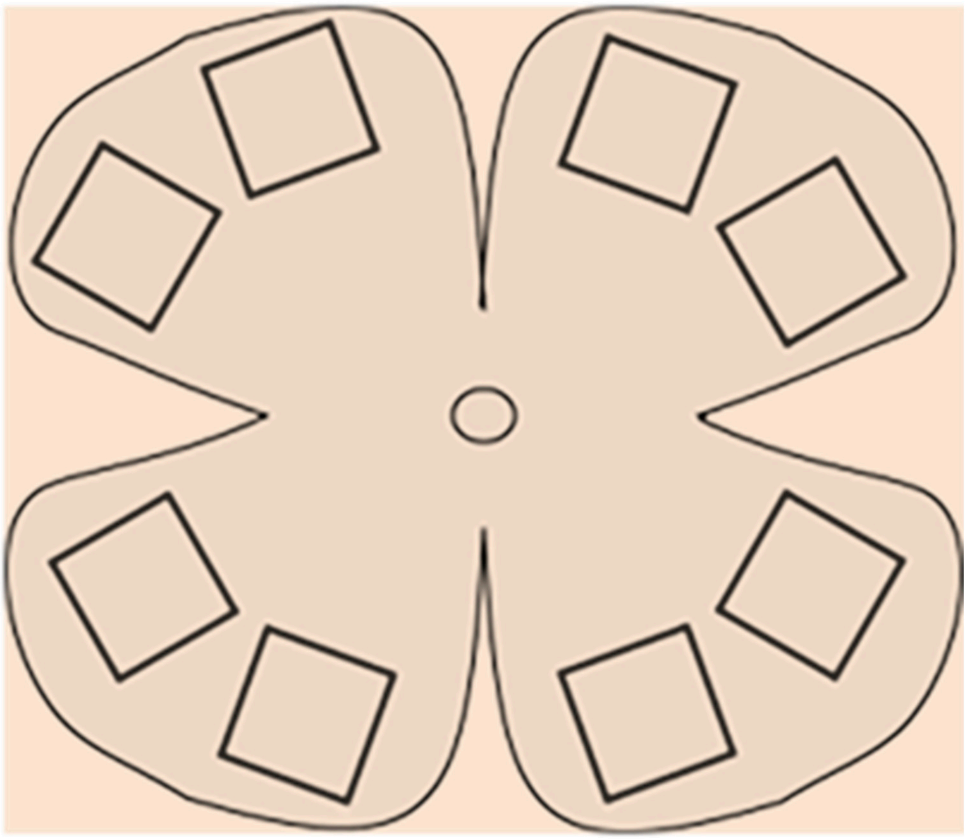

Supplementary figure S4: Illustration of the image position of retinal wholemount analysis on retinal ganglion cells.

For each retina, total 8 images were obtained at a fixed position (boxes) in peripheral retina.

Supplementary Table S1: Primers for gene expression analysis.

Genes		Sequence (5' > 3')	Annealing Temp (ºC)	Amplicon size (bp)	References
Csrp3	F:	CAGCAGTGAGCTGAACAAGTTAC	60	117	NM_013808.4
	R:	CTCCACATTTTGCACCTCCAC			
Glb1l3	F:	TGGAAGGACCGCTTGTTGAA	60	169	NM_001113323.1
	R:	GAAGAATCACCCACAGCCCA			
Hdc	F:	TGTGAATACCGTGAATACCGTGA	60	200	NM_008230.6
	R:	ACTCGTTCAATGTCCCCAAAGA			
Kif4	F:	ACAGAGAAGCAGCTGGAGGA	60	181	NM_008446.3
	R:	TCTGTTTGCTGGCTACTTGGA			
Cfh	F:	ACAGGAGAACAAGTGACATTCAGA	60	146	NM_009888.3
	R:	GTAGCATTTGGCACATGTGGTG			
Chi3l1	F:	TCCAACACTGAGAGACGCAC	60	170	NM_007695.4
	R:	GGCTGGACCTCCTTTGTGAA			
Cntn1	F:	CCGCCTAAGCGAGGAGTG	60	149	NM_007727.3
	R:	GGTAAATGCAGTTGACGCTGT			
Cp	F:	CATTGCTGCTGAGGAGGTCA	60	122	NM_007752.4
	R:	TTCTTGTGGCACCTTGCTCA			
Cyp1b1	F:	GCCTGCCACTATTACGGACA	60	134	NM_009994.2
	R:	CCACAACCTGGTCCAACTCA			
Edn2	F:	TTTCGTCGATGCTCCTGCAA	60	200	NM_007902.3
	R:	CGATGGCAGAAGGTAGCACA			
Fgf2	F:	GCTTCTTCCTGCGCATCCA	60	176	NM_008006.2
	R:	ACACTTAGAAGCCAGCAGCC			
Impg2	F:	GGAGTTTTGCTCCCCCAAAC	60	173	NM_174876.3
	R:	GGAGAACTGGAGCTGAAACCA			
Jak3	F:	AAGACCCGGATAGCAGCCA	60	130	NM_010589.6
	R:	GCTGAGAAGCCTGGAGAGGA			
Marcks	F:	TTTCTTTGTTGAAGAAGCCAGCAT	60	148	NM_008538.2
	R:	GTTCACTTTTACGTGGCCATTCTC			
Pcdh7	F:	TCCGAGTACAGCTGTCAAACAA	60	138	NM_018764.3
	R:	CGTAGAAGCAAGGTGAAGACAGA			
Pmel	F:	AGCAACAACCACAGAGGGTC	60	163	NM_021882.5
	R:	GGCGAGGGAGAAAGAACCAT			
Serpina3n	F:	AGCTATCACAGAGGCTGGCA	60	156	NM_009252.2
	R:	GACTGCAGCATCCATTCCCAA			
Sparc	F:	ACCTGGACTACATCGGACCA	60	195	NM_009242.5
	R:	CCAGGCGCTTCTCATTCTCA			
Actb	F:	GCTCCGGCATGTGCAAAG	60	100	NM_007393.5
	R:	CCCACCATCACACCCTGG			

Supplementary Table S2: Differentially expressed genes in the retina of the 16-month-old mice compared to that of the 2-month-old mice.

Gene name	Average FPKM (16-months)	Average FPKM (2-months)	log2 Fold change	P_{corr}
Gm19441	25.61	0.00	7.01	1.00×10^{-5}
Gm7239	88.36	1.46	5.94	3.79×10^{-11}
Gm47480	58.08	1.77	5.02	1.42×10^{-8}
Nod2	75.66	4.63	4.02	1.01×10^{-9}
Gm28876	247.08	17.07	3.86	6.09×10^{-26}
Calcr	94.80	6.88	3.79	2.41×10^{-12}
Gsc2	79.49	5.82	3.78	6.00×10^{-11}
Csrp3	129.17	10.10	3.68	7.64×10^{-15}
Gm28265	75.18	6.38	3.56	0.011
Gm10830	25.90	2.54	3.36	0.003
Glb1l3	1722.81	169.01	3.35	1.37×10^{-11}
Tmem139	25.24	2.46	3.33	0.002
2410124H12Rik	98.69	10.16	3.29	9.27×10^{-13}
Gm11843	93.48	9.64	3.27	1.04×10^{-8}
Gm11032	23.06	2.49	3.20	0.003
Gm45132	229.19	27.52	3.05	1.79×10^{-19}
C430014B12Rik	77.77	10.33	2.90	6.28×10^{-7}
Rdh1	88.24	12.35	2.84	9.64×10^{-10}
Gm2027	29.08	4.32	2.75	0.002
C230086J09Rik	41.03	6.15	2.74	3.53×10^{-4}
1700116B05Rik	28.71	4.32	2.73	0.001
Six1	49.75	7.63	2.71	1.00×10^{-5}
Rnf43	76.20	11.78	2.68	3.69×10^{-7}
Hdc	1317.41	218.29	2.59	1.63×10^{-49}
Gm45011	21.56	3.57	2.59	0.011
Gm22009	27.14	4.70	2.54	0.029
Tcea3	95.82	16.64	2.53	3.31×10^{-9}
Tex47	30.99	5.43	2.51	0.007
Nek5	33.50	6.16	2.45	0.004
Gm42772	21.50	3.90	2.45	0.025
Gm26611	37.08	7.20	2.36	0.001
Nr4a3	56.66	11.16	2.34	0.001
Adam1b	118.23	23.43	2.33	1.32×10^{-8}
Col4a3	1816.93	364.95	2.32	7.81×10^{-7}
Gm45182	23.65	4.75	2.32	0.019
Gm5954	31.40	6.46	2.27	0.010
Kif4	445.21	93.13	2.26	3.36×10^{-16}
Rimbp3	235.94	49.87	2.25	1.26×10^{-15}
Gm5478	245.00	51.51	2.25	3.29×10^{-11}
Gm12260	29.33	6.19	2.25	0.016
4930477O15Rik	51.28	10.79	2.24	1.72×10^{-4}

Gm11953	27.16	5.73	2.24	0.015
Gm33497	112.32	23.93	2.23	1.91×10^{-6}
A930014E10Rik	39.08	8.28	2.23	0.007
Ube2t	64.31	13.99	2.20	6.04×10^{-5}
Rn7sk	167.34	36.40	2.20	0.022
AC124739.2	29.73	6.88	2.11	0.009
Shisal2b	93.01	21.80	2.10	6.08×10^{-4}
Gm11961	1768.18	414.38	2.09	1.27×10^{-30}
2810405F15Rik	30.81	7.26	2.09	0.010
Gchfr	28.56	6.91	2.05	0.014
Trim43c	211.06	51.53	2.03	3.71×10^{-11}
Ect2	161.82	39.90	2.02	6.89×10^{-10}
Chil5	42.57	10.95	1.95	0.014
Gm37589	45.92	11.96	1.94	0.002
Ly75	245.93	66.96	1.88	5.38×10^{-12}
AC149090.1	3675.11	1003.85	1.87	7.70×10^{-14}
Gm2396	94.21	25.79	1.86	2.38×10^{-5}
Gm19786	88.28	24.87	1.82	7.98×10^{-5}
Gm48692	35.75	10.07	1.82	0.020
Zfp703	229.95	65.97	1.80	4.52×10^{-10}
1700016P03Rik	70.85	20.46	1.79	0.004
Sox17	26.13	7.58	1.79	0.041
Gm47647	2834.40	849.17	1.74	6.54×10^{-24}
Immp2l	68.73	20.70	1.74	6.08×10^{-4}
Pifo	55.39	16.54	1.74	0.002
Hist3h2a	924.19	289.31	1.68	5.99×10^{-21}
Hist4h4	68.60	21.48	1.67	0.003
4933413L06Rik	61.30	19.24	1.67	0.010
9330159M07Rik	526.25	166.21	1.66	3.73×10^{-13}
Slc29a4	378.83	119.89	1.66	6.43×10^{-6}
Col4a4	894.99	284.57	1.65	3.84×10^{-12}
Gng11	147.98	47.84	1.63	3.57×10^{-5}
Tec	86.61	28.36	1.61	3.24×10^{-4}
Gm45218	46.09	15.07	1.61	0.013
Prok1	110.18	36.26	1.60	1.03×10^{-4}
Isyna1	560.60	188.93	1.57	1.61×10^{-15}
Tnxb	218.16	73.80	1.57	8.40×10^{-6}
Grem2	95.13	32.09	1.57	0.002
Fndc1	1479.10	502.35	1.56	1.61×10^{-18}
Tmem116	261.93	88.97	1.56	2.81×10^{-9}
2410018L13Rik	118.27	40.01	1.56	0.002
Gm44996	39.23	13.37	1.55	0.043
Fam90a1b	38.31	13.40	1.52	0.030
Trim17	720.72	254.30	1.50	3.26×10^{-17}
A130077B15Rik	62.43	22.04	1.50	0.012

Ceacam1	59.06	20.99	1.49	0.005
5730507C01Rik	97.78	35.21	1.47	0.002
Gm10654	63.63	22.93	1.47	0.004
Misp	49.83	18.35	1.45	0.038
Zbtb8a	205.43	77.19	1.41	2.36×10^{-6}
Itpr3	82.29	31.33	1.39	0.002
Gm49422	51.33	19.80	1.37	0.026
Gm4065	60.70	23.63	1.36	0.018
CT010467.1	27348.67	10726.10	1.35	0.048
Etnppl	335.66	132.93	1.34	6.72×10^{-7}
Ccdc180	247.46	97.79	1.34	7.02×10^{-7}
Hcls1	630.19	248.32	1.34	1.70×10^{-6}
Epb41l4aos	125.04	49.34	1.34	0.002
Gm35256	62.49	24.66	1.34	0.018
Gm9929	121.65	49.04	1.31	6.79×10^{-4}
Slc37a2	164.26	66.63	1.30	6.90×10^{-5}
B230220B15Rik	134.69	54.98	1.29	7.39×10^{-4}
Alpk2	175.34	72.81	1.27	3.49×10^{-5}
Myo15	68.02	28.13	1.27	0.016
Gm11754	313.81	130.66	1.26	1.32×10^{-7}
Gm10699	111.76	46.62	1.26	0.001
Irf6	65.90	27.49	1.26	0.014
Hspa2	79.43	33.39	1.25	0.010
Irs1	165.46	70.13	1.24	0.001
Hfe	383.22	163.81	1.22	4.95×10^{-6}
Stard8	55.98	24.08	1.22	0.043
Gm7628	49.24	21.35	1.21	0.050
Cyp27a1	158.72	69.40	1.20	2.84×10^{-4}
Gzmm	336.41	148.96	1.18	7.28×10^{-4}
Gngt2	401.71	178.04	1.18	0.002
Gm13136	69.01	30.53	1.18	0.046
Zim1	283.02	125.38	1.17	1.03×10^{-4}
AC163634.1	88.10	39.18	1.17	0.010
Retn	97.83	43.39	1.17	0.011
Rncr4	1238.09	555.60	1.16	1.46×10^{-4}
Hif3a	178.06	79.87	1.16	0.002
2410006H16Rik	117.71	52.82	1.16	0.044
Npas4	147.02	66.23	1.15	0.009
Snrpg	176.05	79.34	1.15	0.011
Racgap1	234.81	106.17	1.14	1.95×10^{-5}
Clic6	264.66	120.82	1.13	2.16×10^{-5}
Gm49329	82.95	37.99	1.13	0.020
Mapk15	90.00	41.19	1.13	0.036
Sik1	122.98	56.13	1.13	0.036
Asb2	73.75	34.18	1.11	0.033

Prrt4	73.14	33.89	1.11	0.036
Gm29595	97.95	45.68	1.10	0.014
Hist3h2ba	487.69	229.50	1.09	1.25×10^{-4}
Gm14091	161.87	76.35	1.09	0.003
Cpeb1	861.92	407.46	1.08	9.06×10^{-7}
9030624G23Rik	152.18	72.15	1.08	0.008
AC114585.1	98.19	46.51	1.08	0.038
Zwilch	60.29	28.42	1.08	0.038
Sbsn	212.96	101.69	1.07	0.001
Glt1d1	138.21	65.91	1.07	0.004
Gm9899	95.71	45.88	1.06	0.009
Gm26642	66.45	32.02	1.05	0.039
Insm1	708.24	346.55	1.03	1.24×10^{-5}
Hist1h1c	968.56	476.97	1.02	2.86×10^{-5}
Chtf18	509.36	250.94	1.02	1.88×10^{-4}
Elk3	135.23	66.92	1.02	0.006
Rpl37rt	170.19	83.97	1.02	0.011
Slc5a5	114.58	56.49	1.02	0.026
Rad51	83.75	41.28	1.02	0.034
Ip6k2	4202.45	2087.33	1.01	2.70×10^{-7}
Gnmt	93.81	46.74	1.00	0.018
Bbs12	528.93	1058.11	-1.00	4.78×10^{-6}
Marcks	497.08	995.44	-1.00	1.04×10^{-4}
Slc35f1	150.04	300.44	-1.00	9.06×10^{-4}
Limd1	102.59	205.76	-1.00	0.002
Gm38253	116.80	233.00	-1.00	0.003
3300002I08Rik	54.32	108.82	-1.00	0.010
Kctd12	47.22	94.70	-1.00	0.019
Gm42727	36.48	73.13	-1.00	0.043
Lgmn	875.42	1757.01	-1.01	3.27×10^{-7}
Tuba1b	2312.31	4655.25	-1.01	8.49×10^{-5}
Cckbr	51.01	102.36	-1.01	0.033
Fzd10	57.88	116.85	-1.01	0.041
Ano3	144.85	293.18	-1.02	1.76×10^{-4}
F8	68.52	138.44	-1.02	0.002
Mmp9	48.03	97.40	-1.02	0.050
Cntn1	682.91	1398.64	-1.03	7.54×10^{-9}
Itga4	1658.13	3375.45	-1.03	1.56×10^{-7}
Igfbp5	189.35	386.00	-1.03	3.53×10^{-4}
Msn	157.27	322.48	-1.03	0.003
Mmp16	49.61	101.37	-1.03	0.016
Bche	51.90	105.49	-1.03	0.016
Vmn2r84	42.73	87.01	-1.03	0.024
Trim38	40.31	82.07	-1.03	0.030
Gm7072	196.74	405.45	-1.04	8.26×10^{-7}

Rab27b	183.49	376.06	-1.04	1.02×10^{-4}
Pcdh7	286.38	587.73	-1.04	1.29×10^{-4}
Gm14295	116.80	239.89	-1.04	1.39×10^{-4}
Cfh	127.02	260.05	-1.04	0.022
Atrx	2752.64	5708.36	-1.05	8.91×10^{-13}
BC005561	272.76	565.27	-1.05	2.36×10^{-7}
B3galt2	1156.46	2415.50	-1.06	1.55×10^{-12}
Zfp960	73.21	152.51	-1.06	6.48×10^{-4}
Lpl	94.73	197.65	-1.06	0.009
Elavl4	140.76	295.66	-1.07	0.002
Trim12c	40.85	85.94	-1.07	0.026
Mest	480.35	1012.06	-1.08	1.55×10^{-6}
Cerkl	332.31	700.48	-1.08	4.52×10^{-6}
Gm47982	95.69	201.66	-1.08	3.76×10^{-4}
Cpne4	65.40	138.63	-1.08	0.012
Phip	1702.29	3634.97	-1.09	1.43×10^{-12}
Cd44	71.51	152.66	-1.09	0.005
Tusc5	109.55	233.26	-1.09	0.017
AC129328.1	189.54	407.30	-1.10	4.29×10^{-7}
Tshz3	79.54	170.63	-1.10	2.19×10^{-4}
Pcdhb11	78.42	168.81	-1.10	0.002
Zfp976	45.50	97.31	-1.10	0.005
Tmeff2	42.24	90.39	-1.10	0.011
Lgi2	68.95	148.28	-1.10	0.015
Cd24a	56.88	122.85	-1.10	0.023
Smc4	312.39	675.35	-1.11	8.82×10^{-10}
Tubb2a	409.59	885.24	-1.11	3.68×10^{-5}
Spidr	86.11	186.22	-1.11	7.03×10^{-4}
Pcdhb16	119.37	256.73	-1.11	0.002
Chrna3	53.85	116.05	-1.11	0.008
Cd151	84.99	183.94	-1.11	0.011
Ifih1	36.97	80.02	-1.11	0.024
6820402A03Rik	35.67	76.35	-1.11	0.026
Zfp638	1650.08	3596.46	-1.12	9.24×10^{-15}
Htra1	302.25	658.34	-1.12	3.67×10^{-7}
Pcdhb17	182.59	397.35	-1.12	6.71×10^{-7}
A330076H08Rik	443.61	966.21	-1.12	8.90×10^{-5}
Prdm1	153.51	335.58	-1.13	2.70×10^{-6}
Bmp3	107.14	235.10	-1.13	1.46×10^{-4}
Slc22a30	94.94	207.63	-1.13	2.47×10^{-4}
A930014D07Rik	105.04	229.63	-1.13	0.001
Fut9	148.26	327.47	-1.14	2.52×10^{-6}
Vcam1	62.70	138.16	-1.14	0.001
Zfp781	40.26	88.74	-1.14	0.016
Zfp292	1567.56	3486.27	-1.15	3.77×10^{-16}

Rbp3	28864.33	64054.18	-1.15	4.42×10^{-11}
Nectin3	322.70	717.14	-1.15	4.04×10^{-6}
Dcdc5	72.33	160.23	-1.15	8.50×10^{-4}
Zfp758	150.48	335.87	-1.16	5.32×10^{-7}
Csf1r	77.89	173.34	-1.16	3.02×10^{-4}
Armh4	64.31	144.17	-1.16	7.78×10^{-4}
Fam19a2	44.85	100.25	-1.16	0.027
Nudt6	29.68	66.53	-1.16	0.043
Zfp442	107.17	240.98	-1.17	1.55×10^{-5}
Ctss	73.78	166.15	-1.17	4.83×10^{-4}
Gm42047	105.89	239.32	-1.17	9.80×10^{-4}
Clca1	62.79	140.52	-1.17	0.005
Gm45884	492.75	1117.78	-1.18	4.52×10^{-10}
Dock11	52.30	118.33	-1.18	0.002
Slc26a7	60.31	135.62	-1.18	0.008
Ccdc88a	679.35	1552.28	-1.19	7.70×10^{-16}
Ncam2	185.54	422.87	-1.19	6.61×10^{-6}
Nell2	108.71	247.60	-1.19	4.19×10^{-4}
Heph	51.20	116.76	-1.19	0.001
Postn	45.17	102.56	-1.19	0.007
Gm45540	34.58	78.81	-1.19	0.018
5430403G16Rik	34.49	78.72	-1.19	0.024
Rev3l	1437.63	3297.01	-1.20	1.73×10^{-15}
Tubb5	1964.05	4510.50	-1.20	3.22×10^{-8}
Gm14325	165.97	380.73	-1.20	3.29×10^{-7}
Myo10	662.17	1526.15	-1.20	2.84×10^{-6}
C230004F18Rik	1202.14	2758.66	-1.20	3.41×10^{-6}
9330132A10Rik	82.86	190.97	-1.20	1.47×10^{-5}
Pcsk5	29.85	68.76	-1.20	0.013
Arhgap5	2069.86	4771.81	-1.21	7.18×10^{-17}
Cyp1b1	47.84	110.79	-1.21	0.002
Nipbl	1867.40	4362.79	-1.22	4.06×10^{-19}
Tuba1c	94.57	220.33	-1.22	9.08×10^{-4}
Gm48350	34.84	81.10	-1.22	0.011
Nrtn	30.95	72.00	-1.22	0.012
Gm37694	24.78	57.80	-1.22	0.025
Apobec2	44.21	104.35	-1.23	0.002
Impg2	4252.08	10027.54	-1.24	2.68×10^{-15}
Sec22c	776.72	1834.46	-1.24	3.07×10^{-8}
Gm8186	29.08	68.72	-1.24	0.019
Rhobtb1	135.08	321.32	-1.25	4.66×10^{-8}
AU041133	137.95	327.77	-1.25	4.92×10^{-7}
Pcdh11x	60.25	143.54	-1.25	0.001
Wwc1	44.92	107.10	-1.25	0.002
Gm44249	46.08	108.92	-1.25	0.003

Agtpbp1	3213.63	7679.43	-1.26	6.39×10^{-7}
Ndst4	37.63	89.92	-1.26	0.009
Tuba1a	1945.85	4668.00	-1.26	0.016
Crym	264.96	640.66	-1.27	4.70×10^{-5}
Selenop	104.14	251.31	-1.27	8.60×10^{-5}
Klhl29	404.16	979.80	-1.28	5.76×10^{-7}
Cd9	195.53	475.50	-1.28	9.20×10^{-7}
Cenpe	43.30	105.62	-1.28	0.002
2310001H17Rik	22.49	54.76	-1.28	0.029
Lcorl	393.57	961.13	-1.29	9.02×10^{-14}
Tubb4b	437.84	1068.12	-1.29	1.26×10^{-8}
Pcdhb10	22.50	55.01	-1.29	0.034
F2r	56.08	138.18	-1.30	3.28×10^{-5}
Gm42697	78.46	192.12	-1.30	8.90×10^{-5}
Gprin3	49.01	120.79	-1.30	3.08×10^{-4}
Mpeg1	39.60	97.65	-1.30	0.005
Serpine2	37.61	93.12	-1.30	0.007
Chml	100.46	248.25	-1.31	2.96×10^{-6}
Gm26944	63.15	156.96	-1.31	3.79×10^{-5}
Sacs	561.64	1403.21	-1.32	7.04×10^{-14}
Gm10033	208.55	520.81	-1.32	1.40×10^{-9}
Otop3	48.06	119.51	-1.32	8.77×10^{-4}
Osmr	127.52	319.46	-1.33	1.97×10^{-4}
Sema3a	51.93	130.83	-1.33	0.001
Gadd45b	53.76	135.78	-1.33	0.009
Cryzl2	37.61	94.81	-1.34	0.017
Bmp15	20.14	50.59	-1.34	0.029
Tagln2	18.18	46.07	-1.34	0.033
Agt	27.84	70.33	-1.34	0.036
mt-Nd4	18429.73	46973.86	-1.35	0.039
Col1a2	27.52	70.76	-1.35	0.046
A930030B08Rik	79.93	205.29	-1.36	2.28×10^{-4}
6430511E19Rik	103.66	266.97	-1.37	5.06×10^{-7}
Il1rapl1	33.71	86.97	-1.37	9.16×10^{-4}
Rorb	2804.66	7294.65	-1.38	1.78×10^{-14}
Clrn1	33.71	87.69	-1.38	6.39×10^{-4}
Gm37150	36.82	95.82	-1.38	7.57×10^{-4}
Pou4f1	119.77	311.36	-1.38	9.13×10^{-4}
Pcdhb12	22.30	58.01	-1.38	0.007
Cadm2	2550.11	6677.15	-1.39	3.42×10^{-16}
Gm47033	22.58	58.93	-1.39	0.009
mt-Nd4l	90.67	240.42	-1.40	0.001
BC024063	102.46	271.25	-1.41	2.57×10^{-8}
Sfrp1	21.84	58.06	-1.41	0.029
Pcdhb15	37.13	99.87	-1.42	3.76×10^{-4}

Gm19667	566.09	1519.13	-1.43	2.71×10^{-14}
Gm14399	118.99	319.13	-1.43	5.35×10^{-9}
Kitl	75.72	204.41	-1.43	3.60×10^{-6}
Pdgfra	72.33	195.57	-1.43	1.25×10^{-5}
Ctsc	76.72	207.43	-1.43	1.44×10^{-4}
Zfp804b	105.54	286.02	-1.44	2.72×10^{-9}
Cdh13	25.06	68.22	-1.44	0.004
Gm26954	1273.85	3488.15	-1.45	9.79×10^{-19}
Gm17586	24.89	68.11	-1.45	0.007
AC098880.2	63.94	176.86	-1.47	1.20×10^{-6}
Tmem176b	106.52	294.57	-1.47	1.05×10^{-5}
Fbn2	27.64	77.33	-1.47	0.008
Gm46218	29.98	83.37	-1.48	4.60×10^{-4}
Mcam	23.09	65.05	-1.48	0.006
Gm26555	19.15	53.54	-1.48	0.006
Gm20515	11.70	32.74	-1.48	0.048
Zfp992	22.14	61.81	-1.49	0.005
Loxl4	23.92	67.08	-1.49	0.013
Cp	1392.31	3939.25	-1.50	1.73×10^{-9}
Dnaic2	45.33	129.07	-1.50	6.25×10^{-5}
Gbp7	45.35	128.17	-1.50	2.47×10^{-4}
Gm45359	34.33	96.66	-1.50	0.002
Chst11	29.69	84.71	-1.51	0.003
Gm44830	13.51	38.51	-1.52	0.029
Gm7435	79.95	230.37	-1.53	5.28×10^{-9}
Kcnd2	43.60	126.09	-1.53	1.18×10^{-4}
mt-Nd2	20955.06	60344.94	-1.53	0.001
Wdr95	21.75	62.55	-1.53	0.005
Zfp982	15.86	45.78	-1.53	0.011
Tubb3	260.19	753.83	-1.53	0.020
Gulp1	237.79	691.15	-1.54	7.18×10^{-17}
Tmem163	41.10	118.81	-1.54	2.62×10^{-4}
2810032G03Rik	23.06	67.00	-1.54	0.009
Zfp973	19.50	56.05	-1.54	0.014
Wwtr1	104.67	308.75	-1.56	1.06×10^{-8}
Gm43488	82.59	243.47	-1.56	2.45×10^{-7}
Zfp518a	337.37	1004.10	-1.57	4.11×10^{-24}
Lrrc2	399.16	1181.45	-1.57	6.51×10^{-10}
Chl1	55.31	164.41	-1.57	3.06×10^{-7}
Gucy1a2	20.44	60.49	-1.57	0.002
Gm37446	10.71	31.63	-1.57	0.042
Gm38162	50.78	152.10	-1.59	2.04×10^{-6}
Col8a1	72.56	217.92	-1.59	3.51×10^{-4}
mt-Nd5	27998.99	85030.11	-1.60	4.28×10^{-12}
Rgs5	164.55	499.92	-1.60	5.44×10^{-8}

Tph2	20.24	61.07	-1.60	0.005
Vim	562.64	1708.53	-1.60	0.005
Laptm5	21.10	63.74	-1.60	0.007
Gm15983	649.80	1971.94	-1.60	0.008
C1qa	16.42	50.08	-1.60	0.021
Slc17a6	251.51	767.91	-1.61	2.43×10^{-10}
Itgb8	346.90	1057.18	-1.61	2.77×10^{-9}
Rp1	14368.50	44370.45	-1.63	1.34×10^{-35}
Gm48236	40.03	124.28	-1.63	2.04×10^{-4}
Samd9l	24.11	74.82	-1.63	0.005
mt-Nd6	7608.45	23920.18	-1.65	2.08×10^{-18}
Znf41-ps	19.73	61.82	-1.65	0.003
AI197445	8.94	27.86	-1.65	0.048
Tubb2b	37.13	117.46	-1.66	5.45×10^{-4}
Lcn2	74.11	236.05	-1.67	8.04×10^{-5}
Gm10925	315.03	1000.08	-1.67	0.010
Cep290	1459.52	4689.53	-1.68	1.41×10^{-34}
Gm37755	64.15	204.58	-1.68	8.03×10^{-9}
Fgf2os	72.98	235.10	-1.69	2.60×10^{-6}
A930033H14Rik	183.07	593.52	-1.70	5.08×10^{-12}
Nes	12.48	40.88	-1.70	0.015
Pld4	14.92	49.04	-1.71	0.006
A2m	133.31	437.68	-1.72	0.044
Clu	1926.85	6409.00	-1.73	5.46×10^{-14}
6430562O15Rik	229.95	765.80	-1.74	4.95×10^{-12}
D430019H16Rik	58.93	197.61	-1.74	2.43×10^{-10}
Gm28373	9.67	32.09	-1.75	0.029
Cx3cr1	22.70	77.25	-1.76	2.25×10^{-4}
Gm45356	8.65	29.44	-1.78	0.035
AC154486.3	24.69	85.06	-1.79	1.86×10^{-4}
Gm10222	24.69	85.45	-1.79	0.003
Ms4a7	9.89	33.97	-1.79	0.039
Jak3	38.68	135.34	-1.80	2.62×10^{-4}
mt-Te	36.09	125.12	-1.80	0.001
Sparc	548.11	2046.02	-1.90	5.76×10^{-15}
Cebpd	38.88	146.09	-1.90	8.90×10^{-5}
Adgrg6	12.46	47.72	-1.92	0.001
Gm49450	8.06	30.30	-1.92	0.016
Gm37008	7.64	29.05	-1.93	0.028
Ppp1r1c	7.62	29.27	-1.94	0.033
Nlrc5	14.44	57.09	-1.98	0.014
Gpnmb	108.88	432.75	-1.99	1.01×10^{-9}
Gm28439	89.67	356.87	-2.00	6.04×10^{-10}
Fgf2	461.62	1841.80	-2.00	3.78×10^{-4}
Ifitm3	46.50	195.21	-2.07	1.90×10^{-6}

C1qc	23.24	101.05	-2.11	8.40×10^{-6}
Gm10475	5.33	23.30	-2.11	0.047
Chi3l1	127.42	558.52	-2.13	0.006
Scube1	15.08	66.59	-2.14	0.002
Lcp1	6.58	30.22	-2.17	0.028
Gm48237	22.98	103.69	-2.18	2.99×10^{-6}
Adgrf3	8.19	38.75	-2.24	0.003
Pmel	21.34	101.13	-2.25	4.81×10^{-4}
C1qb	24.74	119.85	-2.27	1.84×10^{-8}
Gm29260	4.85	23.34	-2.27	0.013
Mir384	4.62	23.03	-2.33	0.020
Ptgfr	4.56	22.95	-2.33	0.045
mt-Co2	937.18	4838.68	-2.37	0.010
Ptprc	8.86	47.73	-2.41	5.97×10^{-4}
Tnnt2	52.44	278.94	-2.41	0.032
Chrnb4	16.09	87.80	-2.46	4.43×10^{-6}
Gm8995	3.99	23.55	-2.57	0.008
Gfap	113.37	686.28	-2.60	0.005
Gm44560	5.31	34.91	-2.74	3.98×10^{-4}
Gm37904	11.21	76.60	-2.77	1.22×10^{-5}
mt-Co3	573.30	3903.94	-2.77	0.033
Edn2	115.59	813.20	-2.81	0.009
mt-Atp8	3.02	22.08	-2.87	0.015
mt-Atp6	504.90	3852.01	-2.93	9.21×10^{-5}
Ccr5	2.75	22.50	-3.04	0.002
Gm13821	10.53	89.67	-3.09	1.00×10^{-5}
Gm2004	5.49	47.20	-3.11	1.52×10^{-4}
Wdr63	4.93	44.07	-3.17	2.19×10^{-6}
Plcz1	3.06	28.82	-3.24	4.13×10^{-4}
Gm47887	5.20	50.65	-3.29	1.01×10^{-6}
Gm39464	2.84	27.45	-3.31	6.46×10^{-4}
CT025671.4	10.26	132.66	-3.68	0.002
Serpina3n	32.70	500.56	-3.93	2.36×10^{-6}
Tmem26	4.01	66.53	-4.07	4.21×10^{-11}
Mki67	1.27	24.09	-4.29	6.18×10^{-4}
Bcl3	2.94	64.53	-4.43	6.72×10^{-7}

FPKM: fragments per kilobase of transcript per million fragments; P_{corr}: corrected *P*-value
